# Modulation of Cortical Activity by Transcranial Direct Current Stimulation in Patients with Affective Disorder

**DOI:** 10.1371/journal.pone.0098503

**Published:** 2014-06-10

**Authors:** Tamara Y. Powell, Tjeerd W. Boonstra, Donel M. Martin, Colleen K. Loo, Michael Breakspear

**Affiliations:** 1 School of Psychiatry, University of New South Wales, Sydney, Australia; 2 Black Dog Institute, Sydney, Australia; 3 MOVE Research Institute, VU University, Amsterdam, The Netherlands; 4 St George Hospital, Kogarah, Australia; 5 Queensland Institute of Medical Research, Brisbane, Australia; 6 Royal Brisbane and Women’s Hospital, Brisbane, Australia; University Medical Center Goettingen, Germany

## Abstract

Transcranial direct current stimulation (tDCS) has been shown to have antidepressant efficacy in patients experiencing a major depressive episode, but little is known about the underlying neurophysiology. The purpose of our study was to investigate the acute effects of tDCS on cortical activity using electroencephalography (EEG) in patients with an affective disorder. Eighteen patients diagnosed with an affective disorder and experiencing a depressive episode participated in a sham-controlled study of tDCS, each receiving a session of active (2 mA for 20 minutes) and sham tDCS to the left dorsolateral prefrontal cortex (DLPFC). The effects of tDCS on EEG activity were assessed after each session using event-related potentials (ERP) and measurement of spectral activity during a visual working memory (VWM) task. We observed task and intervention dependent effects on both ERPs and task-related alpha and theta activity, where active compared to sham stimulation resulted in a significant reduction in the N2 amplitude and reduced theta activity over frontal areas during memory retrieval. In summary a single session of anodal tDCS stimulation to the left DLPFC during a major depressive episode resulted in modulated brain activity evident in task-related EEG. Effects on the N2 and frontal theta activity likely reflect modulated activity in the medial frontal cortex and hence indicate that the after-effects of tDCS extend beyond the direct focal effects to the left DLPFC.

## Introduction

Transcranial direct current stimulation (tDCS) has recently emerged as a promising intervention for affective disorders. Clinical improvements following tDCS manifest as reductions in depressive symptoms [1]–[3], as well as improved cognitive performance in attention and working memory domains [4]. As an emerging therapeutic tool tDCS overall is well tolerated, safe and non-invasive, has few side effects, and does not require delivery with anaesthesia [5]. However there is a relative paucity of knowledge regarding the direct effects of tDCS on cortical activity in patients experiencing a major depressive episode [5], [6], impeding both the development of quantitative markers of physiological response to stimulation and a more personalised treatment approach.

Electroencephalography (EEG) is an obvious candidate for studying changes in cortical activity following tDCS [7]. tDCS is known to affect neural excitability in a polarity specific way and the neuromodulatory effects are widely acknowledged [8]–[10]. In addition to focal changes around the location of anodal stimulation, tDCS may have wide-ranging cognitive and behavioural effects resulting from propagation through brain networks [11]. EEG has been widely used to study the neural correlates in cognitive paradigms such as visual working memory (VWM) [12]–[18], and hence is now used to investigate the modulatory effects of tDCS. These studies suggest a strong direct effect of tDCS on EEG activity in healthy subjects, both at rest [19] and during cognitive tasks [20]–[24]. For example, Keeser *et al.* [7] found increased amplitude of the P2 and P3 components in a working memory task localized to the parahippocampal gyrus. Similarly, Tseng *et al.* [22] found an increased N2 amplitude and memory performance after tDCS to parietal cortex and Van der Hasselt *et al.* [23] show enhanced N450 amplitudes when inhibiting habitual responses of opposing emotionally valent stimuli. Hence, EEG appears to be particularly suited to study widespread cortical activity changes induced by tDCS.

Whilst these studies sought to primarily elucidate the basic neurophysiological impact of tDCS, the findings suggest a role for EEG as a marker or predictor of response in the therapeutic setting [25], [26]. For instance, a case study by Palm *et al.* [27] reported acute effects of tDCS using EEG obtained from a patient with pharmacological therapy-resistant major depression. There are numerous investigations demonstrating changes in EEG following therapeutic tDCS in patients; with epilepsy [28], stroke [29] alcohol dependence [30], chronic pain [31], and also neuropsychiatric disease including major depression [4], [32]. However no sham-controlled studies examining direct tDCS effects on EEG activity in patients experiencing a major depressive episode have been undertaken. For the therapeutic use of tDCS in patients with depression, a continuous current of 1–2 mA is applied up to 20 min via an anode located over the left dorsal lateral prefrontal cortex (DLPFC) [2]. The neurophysiological mechanisms underlying the antidepressant effects of tDCS remain poorly understood, but it has been recently suggested treatment effects may result from modulation of activity in associated areas connected to the DLPFC such as the subgenual cingulate [11].

The widespread effects of tDCS on cortical functioning and disturbances in prefrontal regions can be effectively assessed using a VWM paradigm [33]. Impairments in cognitive functioning are a well-known neuropsychological consequence in affective disorders and VWM is thus a useful tool for exploring these associated cognitive dysfunctions [34]–[37]. Moreover, tDCS to the left DLPFC has been shown to improve cognitive performance in a VWM task [4], [38]. In patients with major depressive disorder, tDCS has been shown to improve cognitive functions such as working memory [2], [4]. Finally, VWM is known to engage a constellation of brain areas, many located in the prefrontal cortex [18]. By engaging these areas potentially modulated by tDCS, task-related EEG responses may register these indirect effects on cortical functioning.

The objectives of our study were to investigate the acute after-effects of tDCS on cortical EEG activity during a VWM task in patients with an affective disorder during a depressive episode. EEG data were acquired from participants enrolled in a double-blind, crossover study testing effects of a single session of active versus sham tDCS. We studied EEG activity during a VWM task, focusing on traditional evoked potentials as well as event-related spectral changes. Based on previous results on tDCS in healthy subjects, we expected that tDCS would improve working memory and modulate the corresponding activity of prefrontal cortical regions through the propagation of stimulation-induced activity through brain networks. Additionally, we hypothesised that assessing task-related EEG activity in a VWM paradigm would allow characterisation of cognitive sub-functions and quantitative neurophysiological markers affected by tDCS in patients experiencing depression.

## Methods

### Participants

Eighteen participants were invited to participate in our study prior to entry into a clinical trial investigating tDCS stimulation for the treatment of depression at the Black Dog Institute, Sydney, Australia (registered at www.clinicaltrials.gov: NCT00763230) [2]. Data was collected from eighteen participants; however only fourteen data sets were used for the final analysis due to artefactual EEG and poor task performance. Informed consent was given in accordance with the National Health and Medical Research Council guidelines and the study was approved by the Human Research Ethics Committee of The University of New South Wales. Written and informed consent was obtained from all participants prior to study enrolment in accordance with the National Health and Medical Research Council guidelines and the Human Research Ethics Committee of the University of New South Wales.

Participants were diagnosed in a semi-structured interview using the Mini-International Neuropsychiatric Interview (MINI) [39], with confirmation in a clinical interview by a psychiatrist. Participants with a DSM-IV major depressive episode and a score of ≥20 on the Montgomery-Åsperg depression scale (MADRS) were included. In two patients, the present episode occurred in the context of bipolar disorder; all others had unipolar depressive disorders. Trained raters assessed mood and functioning during the study using the MADRS and Clinical Global Impression – severity of Illness (CGI-S). Exclusion criteria included: other Axis 1 disorders, alcohol misuse, drug dependence or misuse, neurological disorders, electronic or metal implants, history of heart disease, electronic or metal implants, treatment with ECT in current episode, pregnancy and concurrent treatment with medications shown to modulate the effects of tDCS (benzodiazepines, anticonvulsants, dextromethorphan and pseudoephedrine). During the study five participants were medication free and all others were taking the following antidepressant medications; duloxetine (2), citalopram (1), venlafaxine (1), duloxetine/mirtazapine (1), desvenlafaxine (1), dothiepin (1), mirtazapine (2), escitalopram (1), olanzapine/paroxetine (1), fluoxetine/zolpidem (1), nortriptyline/lithium (1). For clinical and ethical reasons, participants were not required to withdraw from these medications and any concurrent psychotropic medications were continued at stable doses.

### tDCS

**Figure 1:**
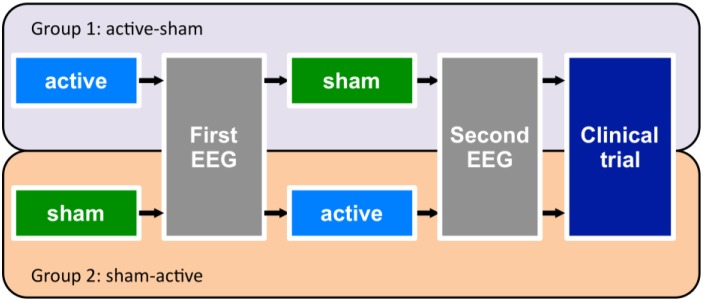
Study design. Each participant received one session of active tDCS (20 minutes at 2 mA) and one session of sham stimulation. The order was counter-balanced across participants. EEG activity was acquired after each stimulation session. Following these assessments, participants continued into the clinical trial (dark blue box) described in Loo et al. (2012).

The study protocol was a double-blind sham-controlled crossover design where participants were randomised to one of two arms of the study in which they received either tDCS followed by sham (Group 1) or sham followed by active tDCS (Group 2) in a ratio of 1∶1 (Fig. 1). Hence, each participant received one session of active and one session of sham tDCS. The tDCS was administered using an Eldith DC-stimulator (NeuroConn GmbH, Germany). The anode placed over the left DLPFC (identified as F3 on the international 10–20 EEG system) and cathode placed over the lateral aspect of the contralateral orbit (at the F8 position). Active stimulation was given at 2 mA for 20 min, with a gradual ramp up/down of the current over 30 s. For sham stimulation, a 1 mA current was applied for 30 s with ramp up/down over 10 s, giving an initial sensation of tDCS while minimising stimulatory effects. Active and sham sessions were performed 7–8 days apart. Following each intervention, EEG activity was acquired to investigate the acute effects of the brain stimulation on cortical activity. EEG data from four participants were excluded because of failure to perform the VWM task at higher than chance levels (1 participant), or because of excessive EEG artefacts (3 participants). Demographic and clinical details of the remaining 14 participants are shown in Table 1.

**Table 1:** Demographic and clinical information.

	*n*	Mean	SD
Gender, male/female	7/7		
Age		40.4	9.67
MADRS		30.9	6.24
CGI		4.64	0.75
Age of onset		28	9.14
Current episode (months)		24.4	26.6
All prior episodes (months)		58.6	61.8
QIDS-SR		16.1	3.9

SD, standard deviation; MADRS, Montgomery-Åsberg Depression Rating Scale; CGI, Clinician Global Impression; QIDS-SR, Quick Inventory of Depressive Symptomatology.

### EEG and VWM Task

**Figure 2:**
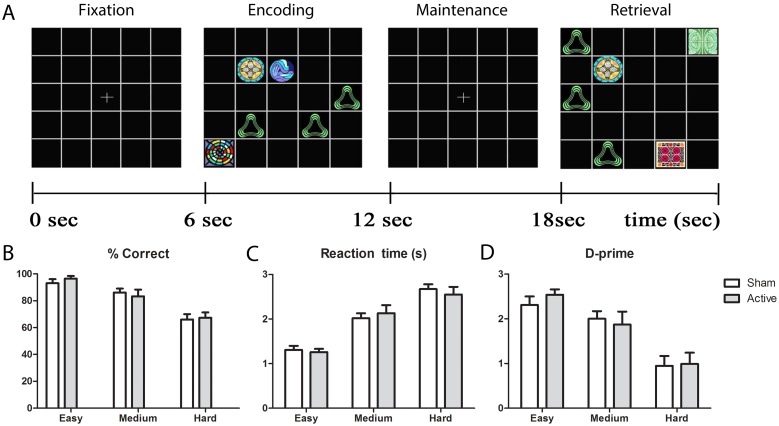
Experimental paradigm and behavioural measures following active or sham tDCS. **A:** Schematic representation of visual arrays presented at regular intervals of 6(0–6 s), encoding (6–12 s), maintenance (12–18 s) and retrieval (18–24 s) screens at varying levels of memory load consisting of easy (1 target, 5 fillers) to medium (3 targets, 3 fillers) and hard (5 targets, 1 filler). This example depicts a true positive trial. **B:** Average proportion correct response across all difficulty levels. **C:** Average reaction time across all difficulty levels. **D:** Average measures of sensitivity scores across all difficulty levels. Error bars show SEM.

Participants were seated in a light and sound attenuated room and completed a VWM paradigm [18]. A delayed match-to-sample task of graded cognitive difficulty was employed. Participants were initially shown a fixation cross for 6 s followed by an encoding screen consisting of a combination of target pictures and non-descript background fillers on a background for 6 s (Fig. 2). The first stimulus was followed by a maintenance screen with a fixation cross that was presented for 6 s (retention phase) and finally a retrieval screen was presented, consisting of the same number of target pictures and background fillers as the encoding screen. Participants were instructed to remember the pictures and positions they appeared in (targets) and responded using custom made buttons. The right button was pressed if one of the pictures appeared in the same locations on the grid, or the left button was pressed if none of the target pictures appeared in the same location. Hence, participants had to remember both the pictures as their locations [18].

The stimuli consisted of a 5×5 grid on which pictures and filler items were presented. Picture stimuli consisted of abstract, multi-coloured designs obtained from an online database (Barbeau, E.J.: http://cerco.ups-tlse.fr/~barbeau/, accessed November 2005), which do not lend themselves easily to verbal naming. Images and locations were randomised to ensure an even presentation of true and false outcomes. By manipulating the ratio of target pictures to background fillers, *memory load* can be parameterised from easy (1 target, 5 fillers) to medium (3 targets, 3 fillers) and hard (5 targets, 1 filler) whilst keeping the visual load and visual scan path constant. Participants undertook 12 trials of each level of difficulty with a total of 36 trials. The order of trials was counterbalanced across subjects.

### Data Acquisition

Scalp EEG data were acquired from 64 channels using BrainAmp MR Plus amplifiers (Brain Products, Munich, Germany, hardware bandpass filter 0.1–250 Hz, resolution 0.1 uV, range +/−3.3 mV) and custom electrode caps (Easy Cap, Falk Minow Services, Herrsching-Breitbrunn, Germany) arranged according to the international 10–20 system. All data were referenced against an electrode centred on the midline between Fz and Cz and sampled at 5 kHz. Electrodes impedances were set below 5 kΩ. The electrooculogram and two electrocardiogram channels were also recorded. The delay between tDCS and the EEG acquisition – due to placement of the cap – was approximately 60 minutes.

### Data Analysis

Task-related EEG data were characterized by conventional ERP analysis to investigate time-locked changes in scalp voltage, and by time-frequency decomposition to assess changes in oscillatory activity that reflect event-related synchronization (ERS) and desynchronization (ERD) of a cortical population [40]. These dependent variables were used to assess modulations of cortical activity by tDCS by comparing task-related EEG activity after active and sham stimulation.

EEG data were analysed using Brain Vision Analyzer software (version 2.0.3). Data was segmented, down sampled to 2 kHz and filtered using a Butterworth zero-phase high-pass filter (cut-off frequency 0.5 Hz). To remove artefacts, EEG data was decomposed using independent components analysis (ICA) using the infomax algorithm. Components containing eye, heart or muscle artefacts were rejected and the remaining component were back-transformed to obtain artefact-corrected EEG data. Cleaned EEG data was re-referenced to the average reference and filtered using a low-pass 45-Hz Butterworth zero-phase filter.

### Event-related Potentials

ERP analysis was used to assess differences between active and sham tDCS conditions on cognitive manipulations relating to memory load. EEG data were aligned to the stimulus onset of the encoding (t = 6 s), maintenance (t = 12 s) and retrieval phase (t = 18 s), and averaged across trials. Grand averages over 14 participants were subsequently calculated and ERP components were labelled as P1, N2, P3 and slow wave corresponding to the time intervals of 90–160, 160–250, 250–400 and 400–800 ms, respectively. The average amplitudes (voltage deviations) over these time intervals were computed and analysed for statistical significance.

### Spectral Analysis

Wavelet decompositions were used to characterise the event-related changes in spectral power during the VWM task. Event-related synchronisation and desynchronisation (ERS/ERD) in identified frequency bands were subsequently computed for statistical comparison across conditions [41]. We employed a continuous complex Mortlet wavelet transformation with 20 frequency steps in frequency range of 1–20 Hz. Baseline correction was performed using the 0–2 s time interval (first 2 s after presentation of fixation cross; see Fig. 2) as baseline period. Spectral power was expressed as relative changes against the baseline period. We then examined task-related spectral changes (ERS/ERD) in the theta and alpha frequency bands following previous research from our group showing strong task-related effects in these frequency bands [40]. EEG data were band-pass filtered in the theta (4–8 Hz) and alpha band (8–12 Hz) respectively and the power in both frequency bands was computed. ERS/ERD were based on the instantaneous power values averaged across 12 trials and spectral power was then averaged over a time interval of 6–7, 12–13 and 18–19 s for encoding, maintenance and retrieval, respectively, and analysed for statistical significance.

### Statistical Analysis

A 2×3 repeated-measures ANOVA was conducted on all dependent variables (behavioural responses, ERPs and spectral changes) with the within-subject factors *intervention* (active or sham tDCS) and *memory load* (easy, medium, hard). For the behavioural data, the percentage correct responses, i.e. hits and correct rejections, reaction times, and d-prime were compared across conditions to assess intervention effects on cognitive performance. All ERP and ERS/ERD components were identically analysed using a 2×3 ANOVA with statistical significance set at p<0.05. The ANOVA was repeated for multiple EEG channels, task conditions and time intervals and significance threshold was adjusted for multiple comparisons using a false discovery (FDR) approach that accounts for the number of conditions and includes a correction for their correlations [42]. The intervention effects on ERPs were tested for the components P1, N2, P3, and the late slow wave for encoding and retrieval. We here focus on channels PO8, Pz and FCz, following our previous work on the effect of VMW whilst recording EEG in healthy subjects [40]. The adjusted significance threshold for the ERP analysis was determined as p<0.0042. The intervention effects on theta and alpha power were assessed in channels PO8, Pz and FCz, for encoding, maintenance and retrieval. The significance threshold for the spectral changes was adjusted to p<0.01. Post-hoc pairwise t-test was performed to determine differences between difficulty levels easy, medium and hard trials for intervention.

## Results

### Behavioural Data

Behavioural data showed significant effects of *memory load*, consistent with the task design. Reaction time (Fig. 2C) increased (*F*(2,26) = 75.6, p<0.0005)) and accuracy decreased (*F*(2,26) = 22.9, p<0.0005) with increasing *memory load* (Fig. 2B). In addition, d–prime (Fig. 2D) decreased as task difficulty increased (*F*(2,26) = 19.68, p<0.0005). There were no significant main effects of *intervention* (active versus sham tDCS) on response accuracy, reaction times or d-prime (p>0.7). No significant interaction effects were observed.

### Event-related Potentials

**Figure 3:**
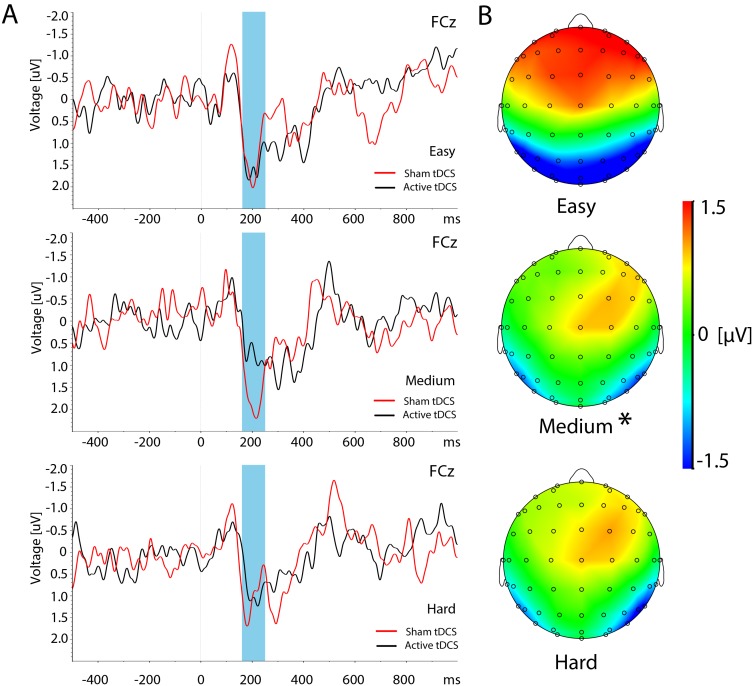
Event-related potentials. **A:** Grand average ERPs of the VWM task during retrieval in channel FCz during easy, medium and hard memory loads (from top to bottom respectively) following either sham (red line) or active tDCS (black line) interventions. Note that the N2 amplitude in channel FCz has a positive polarity due to polarity flipping at frontal electrode sites **B:** Corresponding topographies for the N2 component (160–250 ms) during retrieval after active tDCS. Asterisk denotes the conditions that show statistically significant intervention effects as revealed by post-hoc pairwise t-tests.

Event-related potentials showed waveforms consistent with visually evoked activity. Figure 3 presents an example of the grand average ERP in channel FCz following presentation of the retrieval (second) stimulus, exhibiting large P1 and N2 components that vary with memory load. The expression of ERPs revealed significant effects of memory load consistent with results of a previous study using healthy subjects [40]. The spatial topology of the N2 in retrieval is shown in figure 3B for each difficulty level revealing negative amplitudes over posterior electrodes grading to a strong positive deflection across frontal electrodes. As task load increases the N2 amplitude is reduced, but the spatial topology remains largely intact.

**Figure 4:**
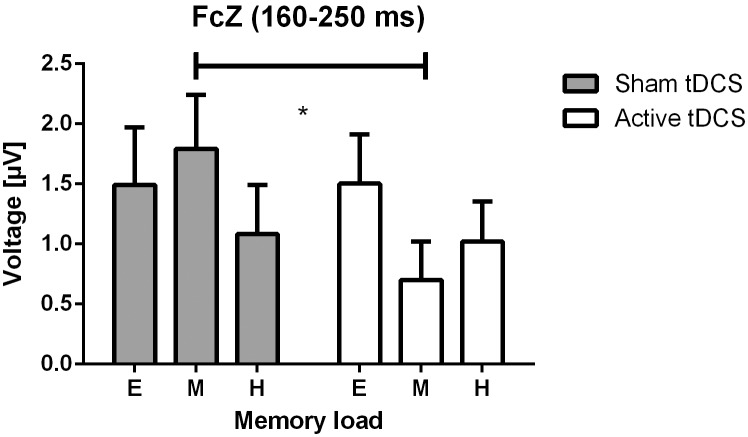
Significant intervention effect on event-related potentials. Bar graph depicts the average amplitude of the N2 component (160–250 ms) in channel FCz during retrieval. White bars show the N2 amplitude after active and grey bars show results after sham tDCS. The x-axis shows the results for different levels of memory load (E: easy, M: medium, H: hard). The interaction effect was significant after correction for multiple comparisons. Pairwise post-hoc t-tests revealed a significant decrease of the N2 amplitude after active tDCS during medium memory load (p = 0.001). Error bars show SEM.

A significant interaction effect of *intervention* and *memory load* on the N2 component during the retrieval interval (18–19 s) was found in channel FCz (*F*(2,26) = 6.87, p = 0.004). Post-hoc t-tests revealed a significant reduction in N2 amplitude after active tDCS compared to sham stimulation in the medium load condition (*t*(13) = 4.01, p = 0.001; Fig. 4). No other significant main effects of *intervention* or interaction effects were found for the event-related potentials.

### Analysis of Task-related Spectral Changes

**Figure 5:**
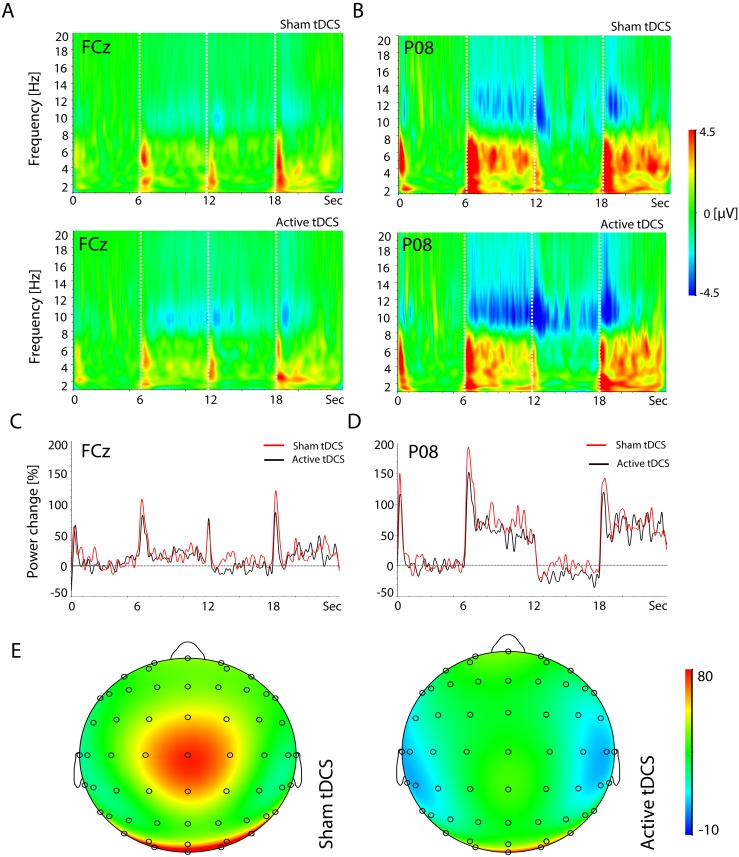
Time-frequency plots of spectral amplitude for the medium task load in occipital and fronto-central channels. **A:** Channel FCz reveals alpha and theta band dynamics across the entire trial in active tDCS (bottom panel) and sham (top panel) interventions. **B:** Channel PO8 reveals alpha and theta modulations across the entire trial in active (bottom panel) and sham (top panel) interventions. **C:** Event-related synchronization/desynchronization in the theta band (4–8 Hz) averaged across all subjects in channel FCz during tDCS active (black line) and sham (red line) stimulation at medium memory load **D:** Event-related synchronization/desynchronization in the theta band in channel PO8 during active tDCS and sham stimulation at medium memory load. **E:** Spatial topographies of event related synchronisation/desynchronisation for the time interval 18–19 s (retrieval) after sham (left panel) and active (right) tDCS.

Visual inspection of changes in the spectral content of the EEG across a broad range of frequencies revealed that task-related changes manifest in the theta (4–8 Hz) and alpha (8–12 Hz) frequency bands (Fig. 5A, B). Spectral amplitude in the theta band increased clearly during stimulus presentation, i.e. during the encoding (6–12 s) and retrieval period (18–24 s), over parietal and occipital cortex. The increase in theta activity (ERS) co-occurs with the ERPs immediately following stimulus onset for encoding (6 s), maintenance (12 s) and retrieval (18 s) both fronto-centrally and occipitally in channels FCz and PO8 (Fig. 5C, D). This increase is then followed by a sustained but reduced power change that continues for the remainder of the interval for each respective task condition, in particular in the occipito-parietal channel PO8. In addition to changes in theta activity, a marked reduction in the alpha activity (ERD) was observed occipito-parietally in the time frequency plots (Fig. 5B). In channel P08 at the start of the maintenance period (12–18 s) there is an abrupt reduction of alpha activity revealed by decreased spectral amplitude. Alpha power revealed a sustained decrease throughout the encoding, maintenance and retrieval periods in occipito-parietal cortex (shown here again in channel PO8). This reduction in alpha activity was stronger at increased memory load, being most apparent in the hard trials.

**Figure 6:**
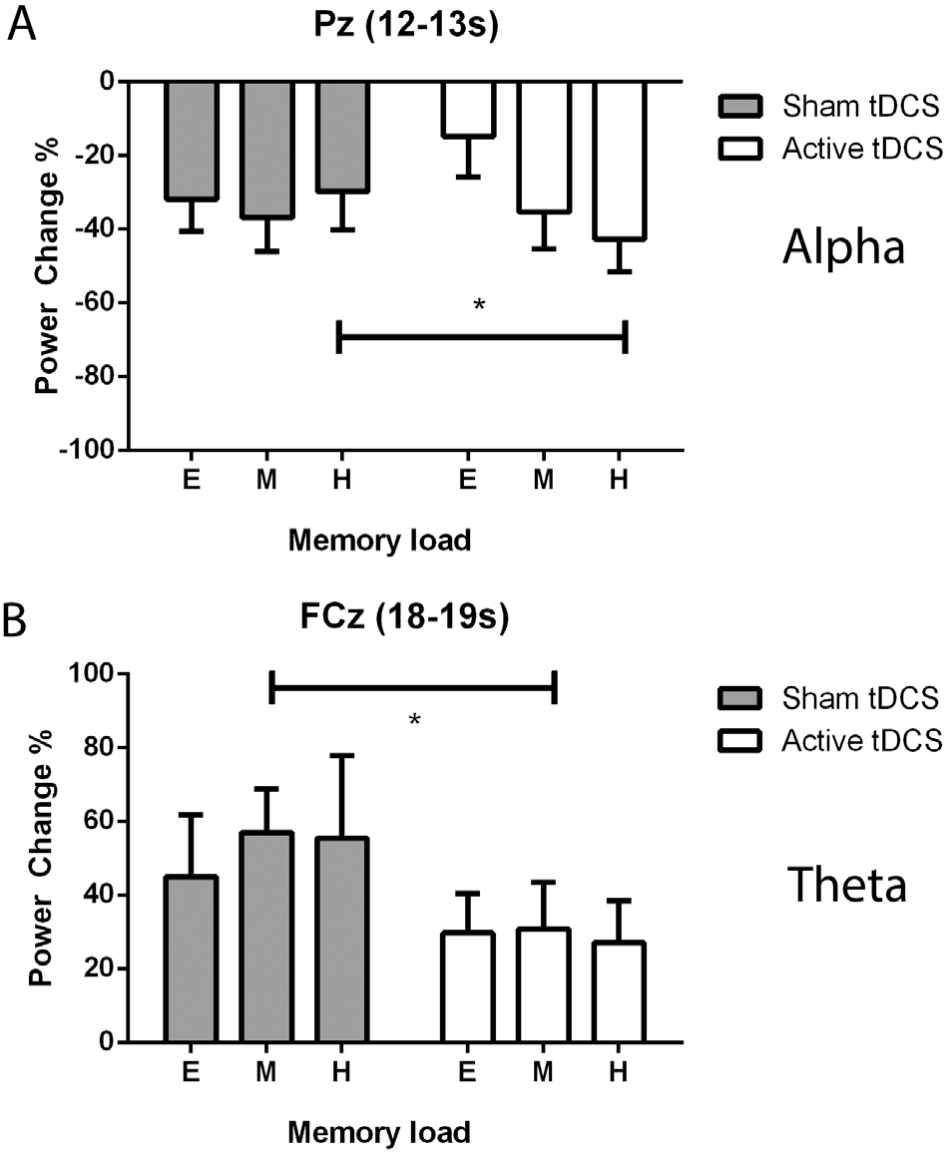
Significant intervention and interaction effects on event-related power changes. Bar graphs depict average changes across subjects in event-related power in parietal and frontal channels after active and sham tDCS. Panel (**A**) depicts results involving significant changes in alpha power, exclusively the interaction between intervention and memory load during maintenance (12–13 s) in channel Pz. Asterisks indicate statistical significance in post-hoc pairwise t-tests, which revealed an intervention effect in the hard memory load condition. Panel (**B**) shows the significant main effect of intervention on theta power during retrieval (18–19 s) in channel FCz. Post-hoc pairwise t-tests revealed an intervention effect in the medium memory load condition. Error bars depict SEM.

Statistical analysis of ERS/ERD data revealed robust tDCS intervention effects on power in both the alpha and theta frequency ranges in frontal and parietal channels (Fig. 6). In particular, a significant interaction effect between *intervention* and *memory load* was observed for alpha power in parietal channel Pz during the maintenance period (Fig. 6A, *F*(1.8,6.2) = 6.23, p = 0.007). Post-hoc t-tests reveal a significant increase in power after active tDCS (*t* (13) = 2.63, p = 0.021) in the condition of high memory load. In addition to the effect on theta activity over parietal cortex, a main effect of *intervention* was observed for theta power in frontal channel FCz during the retrieval period (Fig. 6B, *F*(1,13) = 11.8, p = 0.004). Post-hoc t-tests reveal a significant reduction (*t* (13) = 2.19, p = 0.048) in theta power for the medium task load. No other significant *intervention* or interaction effects were found for ERS/ERD in the theta or alpha band.

### Correlation with Subsequent Improvement in Clinical Trial

After the current study all participants continued with tDCS treatment in a clinical trial to study the antidepressant efficacy over multiple tDCS sessions [2]. Here we sought to perform an explorative analysis to test whether the significant changes in EEG measures found in the present study correlated with the improvements in the subsequent clinical trial. We hence performed a Pearson correlation analysis between the EEG measures that showed a significant intervention effect (N2, Theta and Alpha) and the scores obtained in the clinical trial (MADRS, CGI and SDMT). The participants in our studies were again randomised into an active and sham group when entering the clinical trials. For the correlation analysis, only the participants who received 15 sessions of active tDCS in the clinical trial were used (n = 8). A significant correlation was found between the difference in theta activity after active and sham tDCS and the change in CGI (r = 0.76, p = 0.029) and the change in SDMT (r = 0.81, p = 0.015). However, the effect does not survive removal of an outlier from the data. See Text S1 for further details.

## Discussion

Despite its emerging therapeutic utility the effects of tDCS on cortical activity in patients with an affective disorder remains largely undetermined. Here we examined acute effects of a single session of anodal tDCS to the left DLPFC on task-related EEG activity using a sham-controlled crossover design. We assessed behavioural correlates, ERPs and spectral changes during a VWM task in these patients after receiving tDCS. Respectively we observed significant effects of tDCS on cortical EEG activity in both electrophysiological measures (ERPs and ERS/ERD) during the retrieval period of the VWM task, but found no significant effects on behavioural measures. A distinct finding was that active tDCS resulted in a reduction of the event-related N2 component over prefrontal brain areas during medium memory load. In addition, we observed a significant reduction in frontal theta activity following active compared to sham stimulation and increased occipito-parietal alpha desynchronisation after active tDCS during the retention phase.

To our knowledge, this is the first sham-controlled EEG study investigating the effects of tDCS stimulation in a group of patients with an affective disorder. Our results corroborate previous findings from a case study which recorded EEG after anodal tDCS in a patient with major depressive disorder showing reduction in alpha and theta power [27]. Our findings are consistent with previous EEG studies examining effects of tDCS in healthy participants. The reduction in alpha power over the occipito-parietal cortex after tDCS closely mirrors the reduction in alpha power observed during an emotionally-salient oddball task after anodal tDCS to the left prefrontal cortex [43], during VWM after anodal tDCS to the left prefrontal cortex [24], or the right parietal cortex [20]. The reduction of alpha band activity over occipito-parietal cortices could reflect general effects of tDCS on the fronto-parietal network known to be active when processing stimuli in the VWM system [20]. In addition to reduced occipito-parietal alpha, we also observed a reduction in frontal theta oscillations during memory retrieval in response to tDCS. This finding is supported by previous studies showing reduced frontal theta in resting-state EEG [19], and during a working memory task [44]. A potential explanation of the observed reduction of frontal theta power while VWM performance was unaffected may be that anodal tDCS facilitated more efficient neurocognitive functioning, such that similar performance was attained with less effort. Consistent with the current findings, the effect of tDCS on event related potentials have mainly been reported around 250 ms latency. Keeser *et al.* [7] found increased amplitude of the P2 and P3 components in a working memory task that were localized to the parahippocampal gyrus. Similarly, Tseng *et al.* [22] found an increased N2 (250–320 ms) amplitude and memory performance after tDCS to posterior parietal cortex only in poor performers. Hence, tDCS induces widespread changes in cortical activity reflected by a few key indicators in EEG recordings. The above studies were mostly in healthy subjects and our findings extend this emerging body of knowledge in a clinical population experiencing a depressive episode.

The behavioural effects of tDCS in the literature are mixed and in our study we found no behavioural effects of tDCS on the VWM task. Previous studies have found improved task performance [4], [25], [38], [45], [46], improved accuracy [21] and faster reaction times in response to tDCS [21], [24], [33]. An explanation for the lack of effects in our study may relate to task difficulty: Hard trials may have been too complex resulting in close to chance performance (floor effect), while the easy trials not challenging enough (ceiling effect) [16], [47], [48]. Indeed, the effects of tDCS on the N2 component were only found during medium memory load. In future studies, the sensitivity of the VWM task may be improved by titrating individuals and adjusting task difficulty to individual capabilities. The current paradigm was specifically developed for electrophysiological [40] and neuroimaging [18] studies and may therefore lack sensitivity to detect behavioural changes after tDCS, however there is strong evidence to suggest working memory is a suitable system to characterise the effects of tDCS [33]. In the clinical trial following our study, cognitive performance was measured five minutes after tDCS and improvements were observed [2]. In contrast, in our study a sixty-minute lag time existed between tDCS and the cognitive task due to the EEG setup time and may potentially have diminished the acute effects of tDCS. EEG measures may be more sensitive to the effects of tDCS than behavioural tests, which assess overall task performance and generally involve many interconnected cognitive functions. EEG measures may be able to distinguish between these components and reflect the effect on specific cognitive functions, such as stimulus encoding or attention, as well as distinguishing region-specific effects. This would also further enhance the potential of EEG as an objective clinical endpoint in the research and therapeutic setting.

This exploratory study sought to elucidate the effects of tDCS on EEG measures in view of investigating potential biomarkers for use in a clinical setting. However, in the current study only 18 patients were recruited and four patients were excluded due to high levels of EEG artefacts or inadequate task performance. We chose a within subjects design to optimise the power, given the sample size. The within subjects design is an efficient use of participants time and allowed detection of differences across our different variables with sufficient statistical power to draw inference about the treatment effects. Our study was a satellite study to a clinical trial assessing the efficacy of tDCS in depression [2]. A core feature of the clinical trial was to determine the efficacy for treating patients exhibiting symptoms of depression – symptomatology of both unipolar and bipolar depression. We performed exploratory correlation analysis between the EEG measures and subsequent improvement in the clinical trial (see Supporting Information Text S1). This shows a weak correlation in those participants in whom the regression analysis could be performed (n = 8). However, the effect did not survive removal of an outlier from the data. These early promising findings provide scope for a larger future study that integrates EEG following tDCS and a regression analysis with clinical outcomes, which may disentangle the effects of tDCS in patients with unipolar and bipolar depression. Simultaneous tDCS and EEG [28], [49] would reduce the time required for the EEG set-up and allow for more extensive testing in larger patient groups. Such a study would confirm whether the identified EEG measures indeed predict the differences in clinical improvement after tDCS treatment and thus address questions regarding treatment of affective disorders. Moreover, there is no agreement on what constitutes the most efficacious amount of tDCS, the optimal exposure time [50], [51] or the effect and efficacy of concurrent pharmacological intervention [52]. Potentially, EEG could play a role in monitoring the response to tDCS, using an approach tailored to the more subtle effect of tDCS.

The current findings may also help to uncover potential physiological mechanisms underlying the clinical improvements following tDCS. The observed changes in theta and N2 were found in channel FCz over the medial frontal cortex. The modulation of these EEG components most likely reflects indirect effects of brain stimulations rather than direct effects on the left DLPFC. That is, the frontal theta rhythm in working memory has been associated with the medial frontal cortex [53]–[55]. Likewise, several studies support the view that the N2 component is generated by sources in the medial frontal cortex [56]–[58]. The N2 component is thought to reflect novelty detection and conflict monitoring [59], [60]. This is consistent with the present finding that the N2 is significantly reduced during the retrieval phase when participants decide whether the same stimulus was perceived. The medial frontal cortex is linked to decision uncertainty [61] and also to depression [62], [63]. Moreover, major depressive disorder has been characterized by abnormal patterns in brain oscillations in the theta [64] and alpha band [37]. Hence, the current findings suggest modulated activity of the medial frontal cortex after tDCS engendering the idea that focal brain stimulation propagates through brain networks resulting in wide-ranging effects on cortical functioning [11].

In sum, the present study shows the suitability of EEG to detect the cortical after-effects of tDCS in patients experiencing depression (see also [65]). The observed effects on EEG components are considered to have a source in medial frontal cortex suggesting that tDCS affects cortical functioning beyond focal changes at the stimulation site. Concurrent monitoring of the effects tDCS using EEG would allow mapping these changes in network activity enabling a more personalised approach to tDCS treatment delivery. A neurophysiological understanding of the mechanism underlying the effects of tDCS will further help to optimise treatment protocols.

## Supporting Information

Text S1 **Correlation between EEG measures and subsequent improvement in clinical trial.**
(DOC)
