# Supplementary material for: Modulation of Cortical Activity by Transcranial Direct Current Stimulation in Patients with Affective Disorder
Source: PLoS One. 2014 Jun 10;9(6):e98503. doi: 10.1371/journal.pone.0098503 (PMC4051608; doi:10.1371/journal.pone.0098503)
Supplement: Text S1 — Correlation between EEG measures and subsequent improvement in clinical trial. (DOC) [file pone.0098503.s001.doc]

**Correlation between EEG measures and subsequent improvement in clinical trial**

After the current study investigating the acute effects of a single session of tDCS, all participants continued with tDCS treatment in a clinical trial to study the antidepressant efficacy over multiple tDCS sessions (Loo et al 2012). In the clinical trial participants either received 15 sessions of active or sham tDCS and efficacy of tDCS treatment was assessed before and after the 15 sessions. The MADRS and CGI showed a significantly greater improvement in mood after active than after sham treatment (p<0.05). In the clinical trial, the immediate effects of tDCS on processing speed were also assessed at treatment sessions 1 and 15 (tested immediately before and after stimulation) using the Symbol Digit Modalities Test (SDMT), showing a significant improvement after a single session of active but not sham tDCS (p<0.05). Further details of the clinical trial can be found in Loo et al. (2012).

**Table S1.** EEG measures and mood and cognitive scores from subsequent clinical trial

| **Subject** | **Group^1^** | **EEG Measures** | | |  | **Scores Clinical Trial^2^** | | |
| --- | --- | --- | --- | --- | --- | --- | --- | --- |
|  |  | **N2** | **Theta** | **Alpha** |  | **MADRS***SSRSRS** | **CGI** | **SDMT** |
| 1 | 1 | -2.50 | -4.67 | -4.16 |  |  |  |  |
| 2 | 2 | -0.54 | -7.64 | -28.8 |  | -6 | -1 | 8 |
| 3 | 1 | -1.30 | 4.60 | 1.22 |  | -19 | -1 | 11 |
| 4 | 2 | -2.74 | -132 | -1.06 |  |  |  |  |
| 5 | 1 | -1.99 | -44.0 | -31.6 |  |  |  |  |
| 6 | 2 | -0.528 | -19.7 | -24.9 |  | -12 | -2 | 0 |
| 7 | 2 | 0.254 | -11.2 | -0.048 |  |  |  |  |
| 8 | 1 | 0.521 | 29.6 | 4.10 |  | -8 | -1 | 2 |
| 9 | 2 | -1.86 | -97.0 | -20.2 |  | -19 | -3 | -12 |
| 10 | 1 | -1.45 | -7.85 | -39.9 |  | -9 | -1 | 1 |
| 11 | 1 | -0.036 | -57.7 | 22.6 |  |  |  |  |
| 12 | 1 | -0.762 | 26.6 | -10.9 |  | -13 | -1 | 7 |
| 13 | 2 | -0.489 | -8.83 | -39.5 |  | -2 | 0 | 5 |
| 14 | 2 | -1.872 | -37.4 | -7.65 |  |  |  |  |

MADRS, Montgomery-Åsberg Depression Rating Scale; CGI, Clinician Global Impression; SDMT, Symbol Digit Modalities Test. ^1^In the current study, participants either received one session of active tDCS followed by sham (group 1) or first one session of sham stimulation followed by active tDCS (group 2). ^2^Of the 14 participants that were included in the present study, 8 participants received 15 sessions of active tDCS in the subsequent clinical trial. The other 6 participants received 15 sessions of sham stimulation in the subsequent clinical trial. Correlation analysis between EEG measures and clinical scores was only performed for the 8 participants that received active tDCS in the clinical trial.

Here we sought to test whether the significant changes in EEG measures found in the present study correlated with the improvements in the subsequent clinical trial. This would suggest that the identified EEG measures predict subsequent improvement in mood and cognition after repeated sessions of tDCS. We therefore performed a Pearson correlation analysis between the EEG measures that showed a significant treatment effect (N2, Theta and Alpha) and the scores obtained in the clinical trial (MADRS, CGI and SDMT). Table 1 shows the EEG, mood and cognitive scores for the 14 participants included in this study. For the correlation analysis, only data from 8 participants who received 15 sessions of active tDCS in the clinical trial were used.

**Figure S1. Correlation analysis between EEG measures and mood and cognitive scores obtained during the subsequent clinical trial (n=8).** Three EEG measures were used that showed significant effects of treatment in the current study, i.e. the N2 component in channel FCz during retrieval, theta activity in channel FCz during retrieval and alpha activity in channel Pz during maintenance. For the EEG measures a difference scores was obtained between active tDCS and sham. From the clinical trial the MADRS (Montgomery-Åsberg Depression Rating Scale), CGI (Clinician Global Impression) and the SDMT (Symbol Digit Modalities Test) were used. The change between the MADRS at the beginning and end of 15 sessions of active tDCS was used, while for the SDMT the change before and after the first active tDCS session was used.

Nine pair-wise correlations were hence computed. Figure S1 shows the scatter plots and the corresponding regression lines. There was a significant correlation between the change in theta and the change in CGI (r = 0.76, p = 0.029) and between the change in theta and the change in SDMT (r = 0.81, p = 0.015). These analyses reveal that participants showing higher theta power after a single session of tDCS compared to sham stimulation show a larger improvement on the CGI during the subsequent clinical trial and a larger improvement on the Symbol Digit Modalities Test after a single session of tDCS during the subsequent clinical trial (Fig. S1). While most participants showed an improvement on the SDMT after active tDCS, one participant (participant 9, Table S1) performed worst on the SDMT after one session of active tDCS. This participant also showed lower theta activity after active tDCS than after sham stimulation. This participant appears to be an outlier. We hence repeated the correlation analysis after removing participant 9 (n=7). After removing this participant, none of the correlations between EEG measures and clinical scores were statistically significant.

**References**

Loo, C. K., Alonzo, A., Martin, D., Mitchell, P. B., Galvez, V., & Sachdev, P. (2012). Transcranial direct current stimulation for depression: 3-week, randomised, sham-controlled trial. *The British Journal of Psychiatry*, *200*(1), 52-59.
